# RNA Pol-II transcripts in nucleolar associated domains of cancer cell nucleoli

**DOI:** 10.1080/19491034.2025.2468597

**Published:** 2025-02-23

**Authors:** Soumya Roy Chowdhury, Arunima Shilpi, Gary Felsenfeld

**Affiliations:** a Laboratory of Molecular Biology, National Institute of Diabetes and Digestive and Kidney Diseases; b National Institutes of Health, Bethesda, MD, USA

**Keywords:** Cancer cells, cancer genomics, nucleolus, nucleolus-associated domain (NAD), nucleolus-associated genes (NAG), RNA polymerase II (RNA Pol II)

## Abstract

We performed a comparative study of the non-ribosomal gene content of nucleoli from seven cancer cell lines, using identical methods of purification and analysis. We identified unique chromosomal domains associated with the nucleolus (NADs) and genes within these domains (NAGs). Four cell lines have relatively few NAGs, which appears mostly transcriptionally inactive, consistent with literature. The remaining three lines formed a separate group with nucleoli with unique features and NADS. They constitute larger number of common NAGs, marked by ATAC-seq and having accessible promoters, with histone markers for transcriptional activity and detectable RNA Pol II bound at their promoters. The transcripts of these genes are almost entirely exported from the nucleolus. These results indicate that RNA Pol II dependent transcription in NADs can vary widely in different cell types, presumably dependent on the cell’s developmental stage. Nucleolus-associated genes are likely to be distinguished marks reflecting the cell’s metabolism.

## Introduction

The nucleolus, the largest substructure in the nucleus, is best known as the site of ribosome biogenesis, but in recent years it has become clear that the nucleolus plays many other major roles in cellular function. These include participation in mechanisms of cell cycle progression, the stress response and DNA damage repair, as well as a multitude of other cell processes [1–10].

The chromosomal content of the nucleolus is largely associated with ribosomal RNA biosynthesis. Most extensively studied have been the tandemly repeated ribosomal DNA (rDNA) genes forming the nucleolar organizer regions (NORs), which initiate assembly of the nucleolus following cell division. In humans, the NORs are present on the acrocentric (short p-arm) chromosomes 13, 14, 15, 21, and 22 [11]. The nucleolus has no membrane to separate it from the rest of the nucleus; its compartmental structure is stabilized by phase separation mechanisms [1,4,5] that place actively transcribed ribosomal genes near the center, probably at the boundary between the fibrillar center (FC) and the dense fibrillar component (DFC), and silenced ribosomal genes near the periphery (GC). The nucleolus is not a static structure; regulatory proteins and RNAs cross its boundaries in both directions, in response to signals related to the mechanisms mentioned above, and no doubt to many more as yet undetected (For a review, see [12]).

Over the past several years, there has been increasing interest in the identity and function of genes associated with the nucleolus also characterized as nucleolus-associated domain (NAD) that are not directly involved in ribosome biosynthesis. These genes are embedded in or surrounded by extended regions of nucleolar chromatin. Recent studies have carried out large scale DNA sequencing of purified nucleoli from different human cell lines [13–16], from mouse embryo [17,18], and *Arabidopsis* [19], to identify non-ribosomal gene sequences associated with the nucleolus [12,20,21]. The results have not, in general, identified a unique and common set of NADs, shared across cell types, but they have led to the conclusion that NADs contain genes in which expression is largely silenced in a cell-type specific manner, and are localized to nucleolar periphery for silencing mechanism. Thus, in general terms, chromosomal domains which localize to the nucleolus or nucleolar periphery are termed NADs, which might include repeat elements, heterochromatins, or active genes. The active genes within these NADs are designated as NAGs or nucleolus-associated genes.

It is apparent that any attempt to detect common properties of nucleoli from diverse cell types requires analysis of different cells by similar protocols. Here, we have carried out genomic sequence analysis on purified nucleoli from seven different cancer lines. The genomic location of NADs from different cell lines can vary widely, but three of the cell lines have an unusually large NADs population and share many NAD locations and gene identities. In contrast, the other four have far fewer NADs and have correspondingly few in common, resembling in their properties those previously described [15]. For several of these cell lines we have sequenced the non-ribosomal nucleolar RNA content. In two cell lines, we have also investigated nucleolar genome accessibility by ATAC-sequencing, determined nucleolar sites of RNA polymerase II (RNA Pol II) binding, and mapped histone modifications footprint across the nucleolar genome. The picture that emerges is that in addition to the silenced genes at the nucleolar surface, NADs in some cells may contain transcriptionally active genes marked with activating histone marks, ATAC accessible promoters, and bound Pol II. Interestingly, we find that the transcripts from these active NAD-associated genes are not retained within nucleolus.

## Results

### Isolation of nucleoli and optimization of workflow for nucleolar-associated gene identification

**Figure 1.**
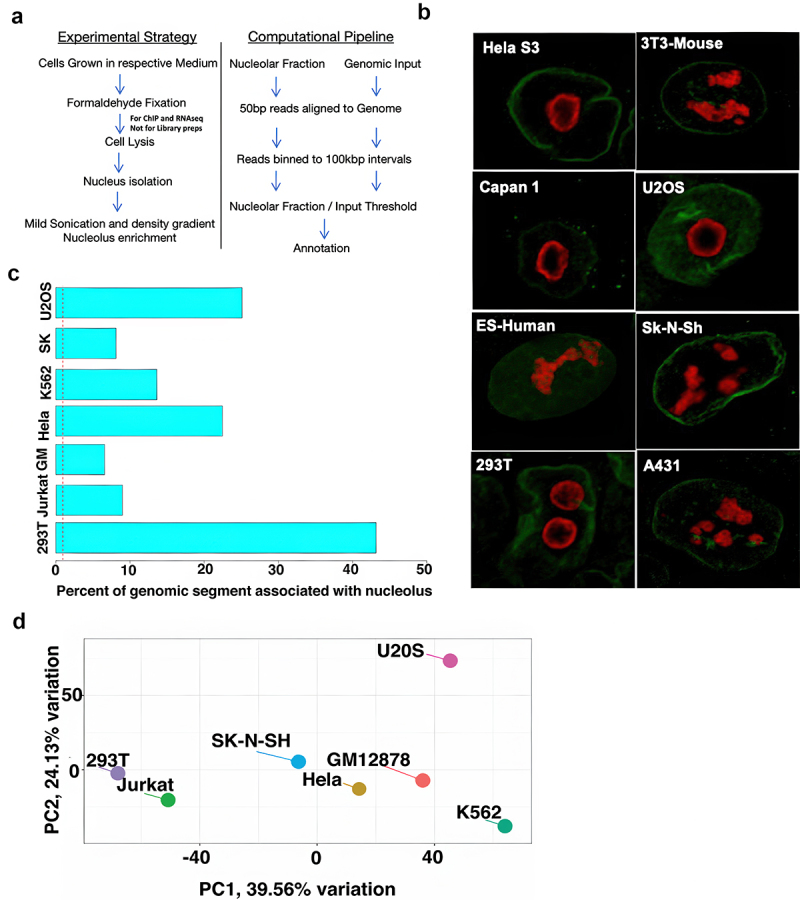
Experimental and computational strategy for determining Nucleolar-Associated Domains (NADs). a) Schematic of experimental strategy for nucleolar purification. b) Representative Immunofluorescence microscopy of nucleolus (anti-nucleophosmin: red) and nuclear membrane (anti-Lamin B1: green) for different human and murine cancer cell lines. c) Bar plot represents the percentage genomic segment associated with the nucleolus in different cell lines d) principal component analysis across different NAD libraries.

To investigate the properties of NADs across cell types, we selected multiple human cancer lines (Table 1). The selection was based on immunofluorescence microscopy to determine the size, shape, and abundance of nucleolar bodies in each cell type (Figure 1(b)). As apparent, though some of the cell types have large and prominent nucleolus as confirmed by microscopy in the representative panel (Figure 1(b)), however, not all could be grown to sufficient quantity for stable nucleolus isolation and NAD studies. In some cases, there were rapid degradation of the nucleolus on isolation resulting in poor yield not suitable for downstream processing.

**Table 1.** Cell lines used in the NAD study.

Cell Line	Morphology	Human Origin
Hela-S3	Epithelial	Human Cervix- Cervical carcinoma
SK-N-SH	Epithelial	Human-brain; derived from metastatic site: bone marrow
U2OS	Epithelial	Human-Bone – Osteosarcoma
K562	lymphoblast	Bone Marrow- Chronic Myeloid Leukemia
Jurkat-BCL2	T Cell/T lymphocyte	Peripheral Blood- Acute T-Cell Leukemia-Male
GM12878	Lymphoblast/B Lymphoblast	Blood. Lymphoblastoid-Female
293T	Epithelial	Human- Embryonic Kidney

Initial analysis across these different cell types revealed that there is limited consistency in the number and size distribution of the nucleoli. However, it was observed that cell lines originating from cancer cells have large nucleolar volume and number per cell, as previously reported for various other human and non-human sources [22–26]. It is well known that nucleolar morphology changes under constraints of physiological condition, metabolic state, RNA polymerase activity, and proteome microenvironment [12,27]. We optimized the nucleolar isolation protocol as previously reported [14,15] and detailed in the methods.

### Overall distribution of NADs across different human cell lines

We identified the NADs associated with each cell type by isolating the total DNA from each nucleolar sample and constructing indexed NGS libraries (see Methods) (Figure 1(a)). The nuclear (genomic) and nucleolar DNA libraries were sequenced, and reads were mapped to the human hg19 reference genome. The count matrix was generated based on the read distribution in 100kbp regions (Figure 1(a)). The nucleolar to genomic ratios were normalized to correct for the background noise. Regions with a relative enrichment of nucleolar (No) to genomic DNA (N) of two-fold or greater were designated as NADs.

The (No/N) fold enrichment ratio in log-scale was plotted for all the chromosomes to map the NADs associated with different cell lines. A wide variation in NAD distribution and abundance was observed across different cell lines. 293T cells have 2059 chromosomal regions classified as NADs, encompassing about 43% of the annotated human genome (Figure 1(c)). It should be noted that this consists largely of gene-free regions. In contrast, the Jurkat-BCl2 and GM12878 lines have fewer NADs but share 69% and 22% of those NADs, respectively, with the 293T cell line. Consistent with their considerable NAD homology, 293T and Jurkat-BCl2 cluster together in principal component analysis (Figure 1(d)). However, cell lines such as Hela-S3, K562, SK-N-SH, and U2OS form distinct group.

**Figure 2.**
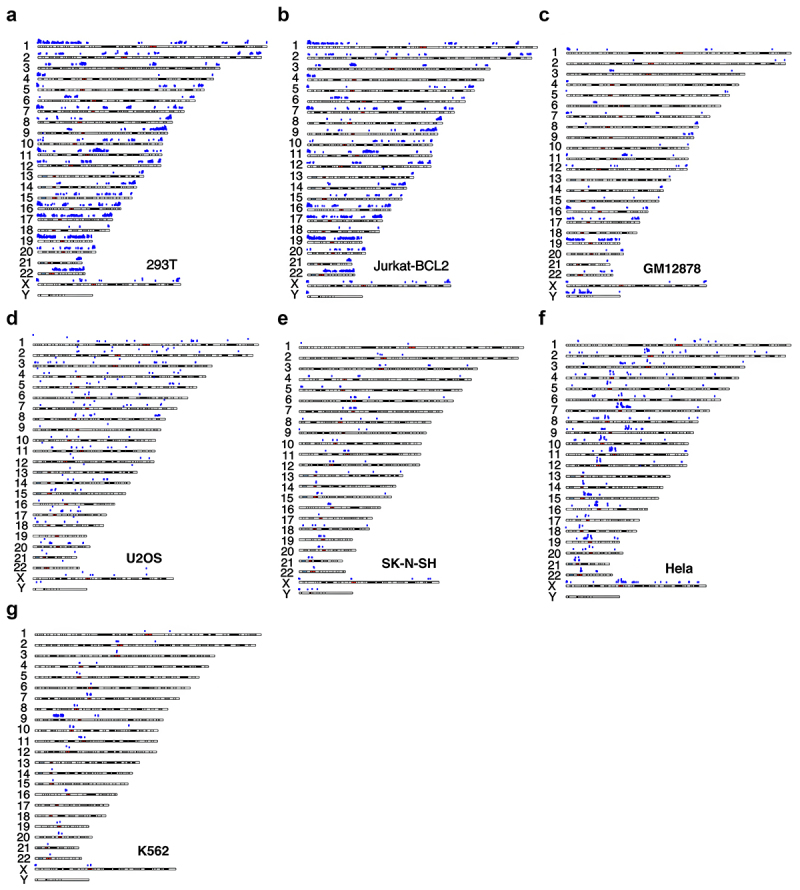
NAD distribution on chromosomes across different human cancer cell lines. Graph showing-karyotypic distribution of NADs in a) 293T, b) Jurkat-BCL2, c) GM12878, d) U2OS, e) SK-N-SH, f) Hela S3, and g) K562 cell lines. The x-axis represents the position along each chromosome and y-axis represents relative enrichment (No/n) in log-scale. The fold enrichment was determined for each bin size of 100 kbp. Each dot in the plot is above the threshold of 2-fold enrichment.

The distribution across chromosomes of the identified NADs in these cell lines is shown in (Figure 2a–g). We found that across these lines, NADs are not confined to acrocentric chromosomes, in agreement with previously reported studies of mammalian (Hela-S3 and HT1080) [13,15] and plant cells (Arabidopsis thaliana) [19] (see Discussion).

There is wide variation in NAD density and distribution on different chromosomes. 293T cells display clustered sites at both the pericentromeric and peri-telomeric regions (Figure 2(a)). GM12878 and Jurkat-BCL2 cell lines show similar distributions (Figure 2(b,c) and S-1B). However, the localization of NADs near pericentromeric regions is more prominent in SK-N-SH, Hela-S3, and K562 cell lines (Figure 2(e,f,g) and S-2C). The pericentromeric mapping of NADs in the Hela-S3 cell line matches the previously published results of Nemeth et al. [15].

We observed common patterns of NAD distribution in 293T, Jurkat-BCL2, and GM12878 cell lines. There are clusters of NADs on some chromosomes, while other chromosomes are almost free of NADs. Clustering on the longer chromosomes is largely confined to 293T cells, which as noted above have the greatest number of NADs. However, all three cell lines show striking concentrations of NADs on chromosome 19 (noted in earlier reports [15]), as well as considerable enrichment of NADs on chromosomes 16 and 17. In contrast, chromosome 13 has very few NAD sites in these lines. This might reflect the fact that this chromosome has the lowest gene density among human chromosomes [28]. Besides acrocentric chromosomes, other of the smaller chromosomes showed higher enrichment of NADs. We observed that in these three cell lines almost the entire length of small chromosomes (chr 16, 17, 19) has a high density of NADs (Figure 2(a,b,c)). In contrast, we observed a more uneven distribution in nucleolar/genomic ratio (No/N) in the larger chromosomes, with clusters of sites near centromeres (chr 6, 11, 12) and telomeres (chr 1, 2,3, 5, 7, 8, 9).

**Figure 3.**
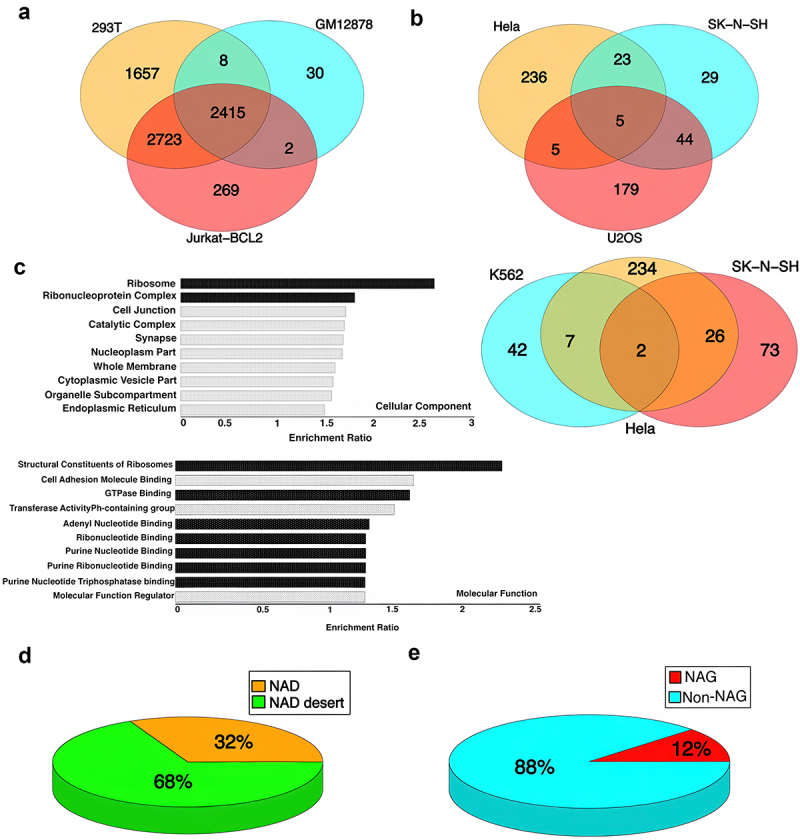
Distribution of nucleolar-associated genes (NAGs) in different cell lines and their functional characterization. Venn diagram showing NAGs common between different cell lines, a) 293T, GM12878, and Jurkat BCL-2, b) Upper: K562, Hela S3, and SK-N-SH cluster and Lower: Hela, SN-K-SH, and U2OS cluster. c) Gene ontology analysis based on 2415 NAGs common between 293T, GM12878, and Jurkat-BCL2 (Functions related to nucleolus or ribosome biogenesis are marked in dark bars). d) Overall distribution of NAD/NAD desert, and e) NAG/non-nag for all cell-lines.

We annotated the NAD-associated genes (NAGs) for each cell line using Entrez gene identifiers [29] (Figure 3(a,b) and S-3). The genes common among two or more cell lines were identified. Surprisingly, 2415 genes were shared among 293T, Jurkat-BCl2, and GM12878 cell lines (Figure 3(a)). Eighty-seven percent of the overlapping genes were associated with protein coding regions, and the remaining 23% were linked to non-coding RNA including snoRNA, miRNA, rRNA, and lincRNA. These genes were further characterized using gene ontology (GO) analysis (Methods) (Figure 3(c)). This analysis shows that in these three lines many genes associated with nucleoli (NAGs) are involved in regulating ribosomal synthesis or functions. In addition, there are genes involved in regulating GTPase-binding, cellular adhesion, transferase activity, and adenyl nucleotide binding. For all studied cell lines, NADs and NAGs constitute only a small subset of the genome (Figure 3(d,e)).

### NAD distribution in chromosome 19

**Figure 4.**
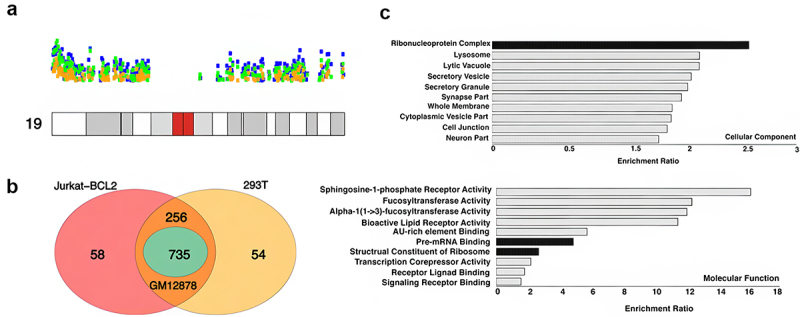
Enrichment of NADs on chromosome 19 and functional significance. a) Karyotypic plot displaying the NADs on chromosome 19 of 293T (blue), Jurkat-BCL2 (green), GM12878 (orange) cell lines. b) Venn diagram showing NAG overlap among 293T, GM12878, and Jurkat-BCL2 cell. c) Gene ontology analysis for 735 NAGs common between 293T, GM12878, and Jurkat-BCL2 cell lines.

Unlike the other human cell lines we studied, 293T, GM12878, and Jurkat-BCL2 cells have an unusually high concentration of NAD clusters on chromosome 19 (Figure 2(a,b,c) and Figure 4(a)). In other cell types in this study (SK-N-SH, K562, Hela S3), chromosome 19’s NAD abundance was comparable to that on other chromosomes. Among the three cell lines with high chromosome 19 NAD density, 293T had the highest number of NADs both on chromosome 19 and genome wide (Figure 2(a)). Chromosome 19 contributes 12%, 16%, and 29% of the total NADs in 293T, Jurkat-BCL2, and GM12878 cell lines, respectively. Smaller chromosomes tend to be enriched in NADs, probably owing to their location closer to the nucleolus [30,31]. Chromosome 19 possesses a high gene density, more than double the genome-wide average [32] which could correlate with the higher NAD density in the three cell lines. However, NAD localization on chromosome 19 is very sparse in the other cell lines (Hela-S3, SK-N-SH, K562) and absent in U2OS (Figure 2(d,e,f,g)), casting doubt on this explanation. Further analysis shows that 2415 NAGs are shared in common among 293T, Jurkat-BCL2, and GM12878 (Figure 3(a)) and that 735 (30%) (Figure 4(b)) are contributed by the chromosome 19 NAD cluster alone.

Gene ontology analysis (Figure 4(c)) shows that for these three cell lines the largest cellular component contribution is from ribonucleoproteins, but contributions from other categories are also observed. However, among molecular functions, pre-mRNA binding and structural constituents of the ribosome are the biggest subset (Figure 4(c)).

We decided to further ascertain these results by performing live imaging of U2OS cells with tagged representative chromosome-19 genomic probes. In this regard, Cas9-mediated fluorescence in situ hybridization (CASFISH) [33,34] protocol was modified and employed using dead-Cas9 (dCas-9) proteins expressed and purified in *E. coli*, which has mutations H840A in the HNH domain and D10A in the RuVC1 domain making then incapable of DNA editing but not in localization to the target genome guided by sgRNA. We designed sgRNA specific to Chromosome-19 genes, as shown in Figure S-6 (A), the choice of the eight targets were random to encompass most of the chromosome-19. Co-transfection of dCas9 with Cy-5 labeled sgRNA into U2OS cell stably expressing NMP-mCherry construct under CMV promoter showed satisfactory evidence that chromosome-19 is in localized to nucleolar boundary and in some cases within the Nucleolus, as shown in the representative images (Fig S-6, B, C, D, E).

### Accessibility of NADs to determine their transcriptional potential

The NADs at the membrane-less nucleolar periphery are known to exist largely as transcriptionally inactive heterochromatin [35–37]. It has been reported, however, that some genes associated with NADs are expressed in the cell lines that have been studied (see Discussion). To address this question, we searched for transcriptionally active NAGs in two of our cell lines, 293T and U2OS, representing the two distinct patterns of NAD distribution described above (Figure 3(a,b)). A major marker for transcriptional activity is accessibility to the cellular transcription machinery. We made use of ATAC-sequencing to search for transposase-accessible sites in the nucleolar chromatin population, modifying the standard ATAC protocol [38] for use on purified nucleoli (Methods).

**Figure 5.**
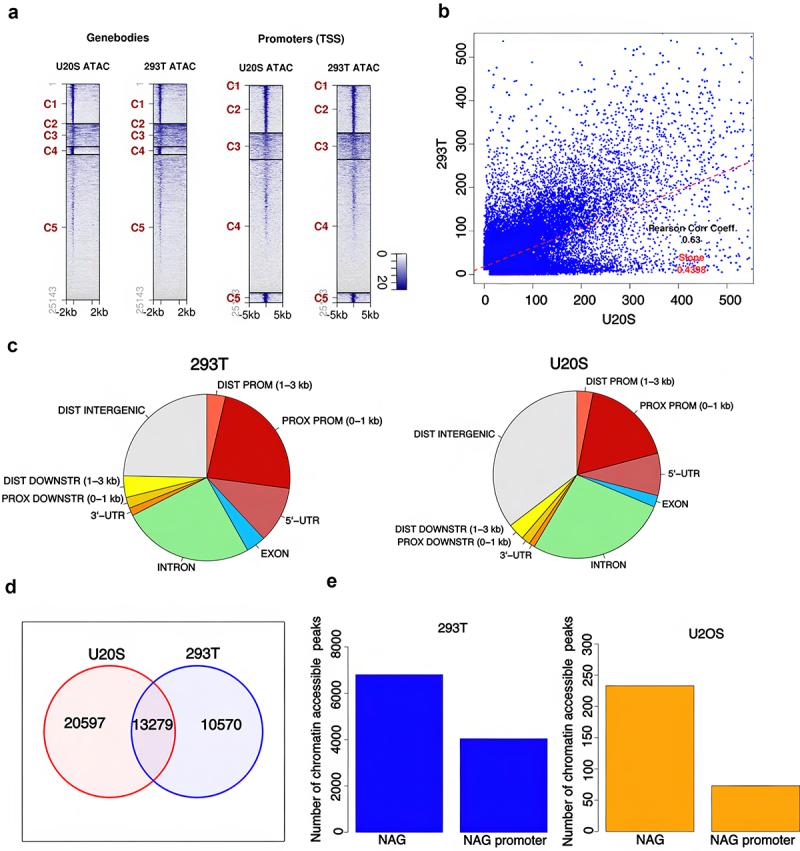
Chromosomal accessible regions in NADs and NAGs of 293T and U2OS cell lines. a) Heatmap showing atac-peaks in the promoter region and gene bodies of the two cell lines. b) Correlation in atac-peaks between two cell lines. c) Annotation of ATAC peaks in two cell lines. d) Venn diagram showing the overlap of peaks between the two cell lines. e) Association of atac-peaks with NAGs and NAG promoters in 293T cell line. f) Association of atac-peaks with NAGs and NAG promoters in U2OS cell line.

The ATAC-seq analysis showed that the NAGs in both U2OS and 293T nucleoli are accessible, prominently in the promoters, promoter proximal regions, and introns of the associated genes (Figure 5(a,c)). We observed a correlation between the peaks in the two cell lines (Figure 5(b)). Despite the divergence in the NAD libraries between U2OS and 293T (Figure 2(a,d)), 39% of U2OS ATAC-peaks overlap with those of 293T (Figure 5(d)). We correlated the chromatin accessible regions with NAGs in the two cell lines. Of the total 23,849 ATAC-seq peaks in 293T cells, 6405 (27%) were associated with the NAGs in that cell line (Figure 5(e)) and of these, 4042 (17%) peaks were associated with promoter regions. However, reflecting lower NAG density, very few ATAC-peaks associated with genes or promoters were identified in the U2OS cell line (Figure 5(e)). Nonetheless, there are many chromatin accessible regions, not part of genes or promoters, associated with U2OS as well as 293T, a considerable number shared in the two cell lines (Figure 5(d)). Overall, these data show that specific regions within NADs could be directly accessible to the cellular transcription machinery and might not be in the facultative heterochromatin state.

### Histone modifications and Pol II binding within NADs

**Figure 6.**
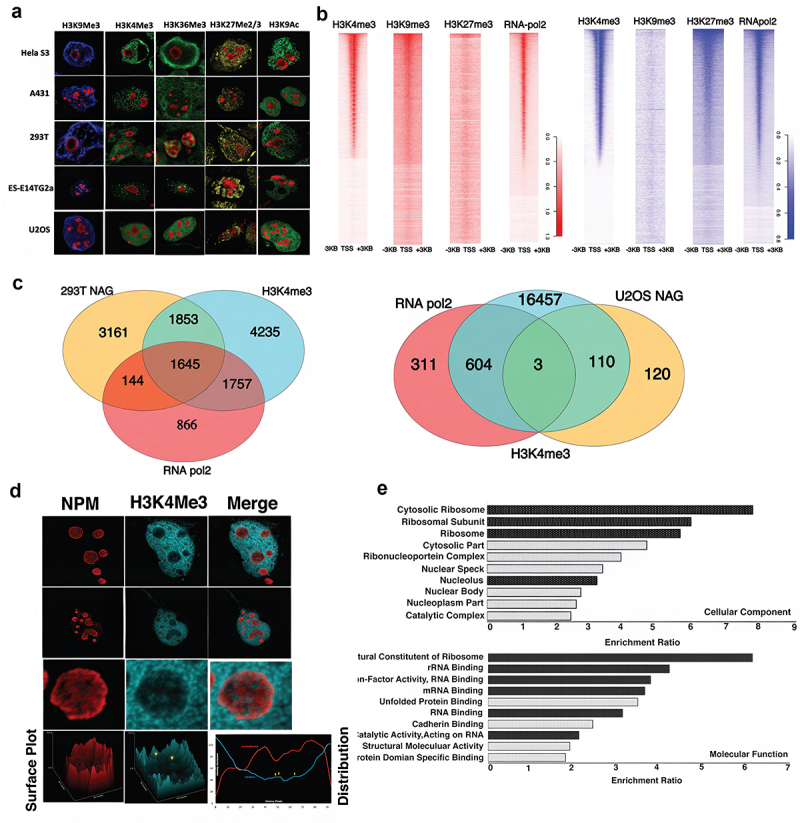
Histone modifications and RNA Pol2 in the nucleolus. a) Double Immunofluorescence (IF) for various histone markers and Nucleophosmin in different fixed mammalian cell types. The histone marks, H3K9Me3 (blue), H3K4Me3 (green), H3K36Me3 (green), H3K27Me2/3 (yellow), and H3K9Ac (green) are represented in columns and the different cell types, Hela S3, A431, 293T, ES-14TG2a, and U2OS are represented in rows. b) Heatmap showing histone marks and RNA Pol2 peaks in the nucleolus of 293T (red) and U2OS (blue) cell lines. c) Venn diagram analysis of overlap in H3K4me3 and RNA Pol2 peaks with NAGs in 293T and U2OS cell lines. d) Double immunofluorescence of fixed U2OS cells with Nucleophosmin (red) and H3K4Me3 (cyan). Surface plot and distribution graph of both the protein shown in lower panel of the Montag showing detachable H3K4Me3 peaks with in the Nucleophosmin boundary of the nucleolus e) Functional characterization of 902 NAGs that are ATAC accessible, have active histone marks and bound RNA Pol2 in 293T cell line.

Given the presence of RNA Pol II associated with the nucleolus and of ATAC-seq accessible sites at NAG promoters, it seemed likely that some or all these sites could be associated with promoters of transcriptionally active genes. To examine this possibility, we used Chip-Seq analysis in 293T and U2OS nucleoli to measure the nucleolar distribution of histone H3K4Me3, a mark associated with transcriptionally active promoters; H3K9Me3, a mark of constitutively repressed genes; and H3K27Me3, associated with facultative heterochromatin (Figure 6a). In both cell lines the silencing marks were relatively sparse, but H3K4Me3 sites were quite abundant (Figure 6b, S-4, and S-5), consistent with the presence of active promoters.

If these promoters are in fact active, they should be associated with RNA polymerase II (Pol II). We therefore carried out Chip-seq studies in 293T and U2OS nucleoli to detect the presence of Pol II. Both cell lines displayed concentrations of Pol II at NAG promoter regions, again consistent with the presence of transcriptionally active genes (Figure 6(b), S-4, and S-5). We compared the number of both H3K4me3 and RNA Pol II peaks associated with the NAGs in two cell-lines (Figure 6(c)). While NAGs in 293T cell-line are associated with 1645 double peaks, only three peaks are associated with NAGs in U2OS cell line. This result was corroborated by our IF results on U2OS where discrete H3K4Me3 pockets were observed within nucleolus boundary as delineated by nucleophosmin (Figure 6(d)).

It then became possible to ask whether there was overlap of the ATAC-seq sites, the Pol II binding sites, and sites carrying the H3K4Me3 modification characteristic of active promoters. As shown in Table 2, 293T nucleoli contain 907 genes sharing all these marks. Gene ontology analysis (Figure 6(e)) shows that there is a strong representation of genes associated with ribosome biosynthesis (see Discussion). U2OS cells, in contrast, had only few such active genes, thus bearing a similarity to other cell lines in which NAGs are largely silenced.

**Table 2.** Summary of NAG accessible to chromatin, histone marks, and RNA-Pol2 in nucleolus and nucleus.

Cell Line	NAD	NAG	Chromatin accessible + NAG	Chromatin accessible + NAG promoter	H3K4me3+NAG	RNA-pol2+NAG	NAG transcript in the nucleus	NAG transcript in the nucleolus
293T	2059	6803	6405	4042	3498	1645	524 (520 protein coding, 4 non-coding)	2
				907 (863 protein coding, 44 non-coding)				
U2OS	281	233	233	73	113	3	2	0
				3				

**Figure 7.**
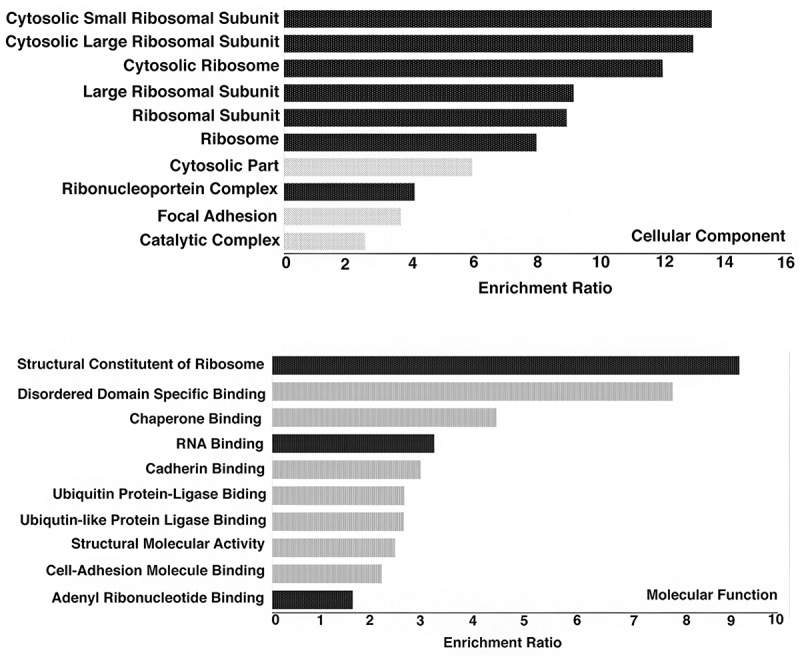
Functional characterization of NAG transcripts found in the nucleus. Gene ontology analysis of 524 NAGs and their cellular components and molecular function.

The presence of 293T cells in this large number of potentially active promoters suggested the presence of corresponding transcripts. We therefore sequenced both nucleolar RNA and total nuclear RNA from these cells and found that 524 NAG transcripts from the active genes were nuclear, but not nucleolar (Table 2). These genes are actively associated with cellular and molecular function of smaller and larger subunit of ribosomes and ribonucleoproteins complex

(Figure 7(a)). These NAGs transported into the nucleus lack repetitive SINE and LINE elements. Thus, the RNAs from the active NAGs identified in the above experiments are being exported from the nucleolus. Of course, the nucleolus also reciprocally imports RNA Pol II transcript [39].

### mRNA and RNA Pol II in the nucleolus

These results raised the question of the transcriptional state of the genes residing in the NADs identified above. It is well established that the nucleolus is principally the domain of RNA polymerase I, necessary for ribosomal RNA biogenesis from the rDNA repeats present in the acrocentric chromosomes; RNA Pol II was thought to be absent or inactive. However, recent studies have detected RNA Pol II activity and the presence of mRNA in the nucleolus [40,41]. Both these results raise questions concerning the contribution of NADs to nucleolar mRNA pools in human cancer cell lines.

**Figure 8.**
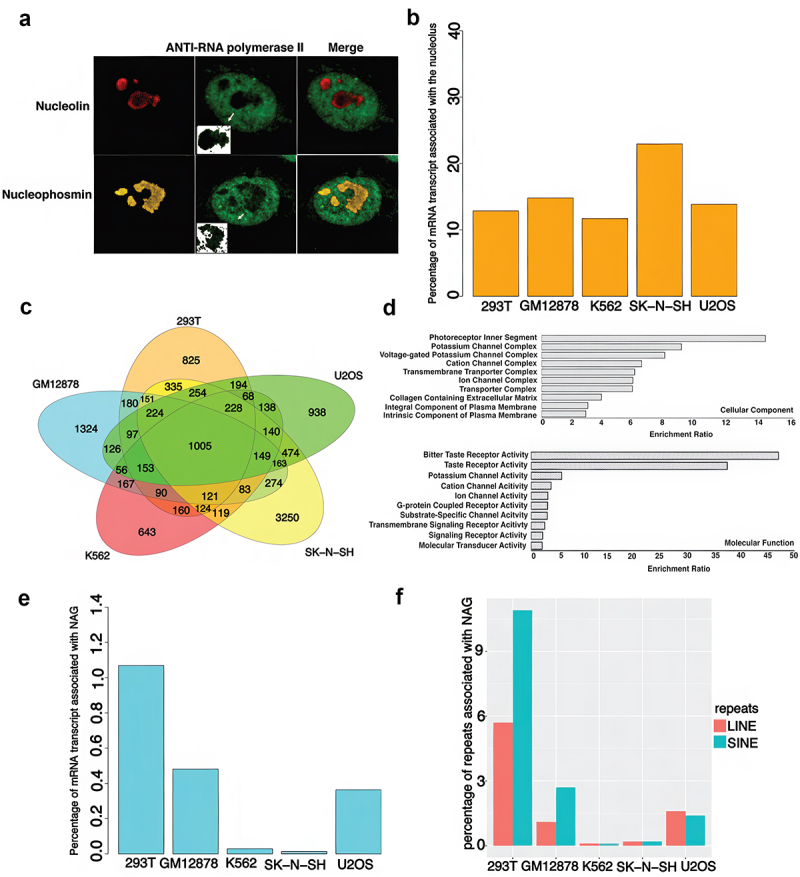
Identification of RNA Pol-2 and Pol-2 genes within the cancer cell nucleolus. a) Double Immunofluorescence (IF) for various nucleolus marker and RNA Pol2 in U2OS cell line, column inset shows the individual nucleolus with the speckles of RNA Pol-2 within nucleolar boundary. b) Bar graph showing the percentage of genes transcribed in the nucleolus in five cell lines. c) Venn diagram analysis of overlap of genes associated with the nucleolus in the five cell lines. d) Gene ontology analysis of 1005 genes common between the cell lines. e) Bar graph showing the percentage of NAGs transcribed in the nucleolus. f) Bar graph showing percentage of repeats associated with NAGs.

To address this, we first performed immunofluorescence microscopy to determine the distribution of RNA Pol II in the nucleolus (Figure 8(a), Fig S7). By counterstaining for Nucleolin and Nucleophosmin we were able to detect the presence of RNA Pol II not only in the nucleolar boundary, but also within the GC compartment. RNA pol-II was more prominent at the nucleolar boundary than within the GC compartment (which is involved in ribosome assembly), where it was specular and diffuse in distribution (Figure 8(a), Fig-S7). This suggests that there is RNA Pol II activity at and possibly within the boundary of the nucleolus, contrary to the earlier literature but supported by recent work mentioned above [39,42,43]. These observations also raised the possibility that the NADs identified earlier in Figure 2 could contain the targets of nucleolar localized RNA Pol II and be the source of nucleolar mRNA.

To address this question directly, we isolated and sequenced total nucleolar RNA (Methods) and identified sequences that were enriched two-fold or more relative to the total cellular mRNA derived from the corresponding cell line. Overall, non-ribosomal nucleolar RNA pools from 293T, GM12878, K563, and U2OS constitute 10–15% of the total cellular mRNA (Figure 8(b)), whereas for SK-N-SH 25% of total mRNA is nucleolar. On average 70% of these RNAs are non-protein coding RNA species, primarily snoRNA, antisense-RNA, miRNA, lincRNA, classes which had previously been reported and characterized as being involved in ribosome biogenesis, function, and regulation [44–46]. However, about one-third of the total nucleolar non-ribosomal RNA pool (from 22% in U2OS to 38% in SK-N-SH) is composed of mRNA transcribed from protein coding genes (Fig S-5).

There is substantial overlap of the non-ribosomal nucleolar RNA fractions among cell lines which show limited overlap of NAD sites mapped at the chromosomal level (Figure 8(c)). We cataloged 1005 protein coding genes, shared among 293T, GM12878, U2OS, K562, and SK-N-SH whose gene products are enriched in the nucleolus and are primarily of non-NAD origin. The gene ontology analysis of these genes is in Figure 8(d). Biological, cellular component, and molecular function analysis of the common gene products reveal no significant attributes shared by these genes that can provide insight into their nucleolar localizat*ion*. However, a significant portion of this group encodes for proteins associated with ion channel complexes.

Interestingly, only a very small fraction of NAGs is being transcribed in the nucleolus (Figure 8(e)). Among the different cell lines, NAGs in 293T cell line constitute 1.2% of the total transcript, whereas K562 and SK-N-SH cells have the smallest fraction of NAG transcript in the nucleolus. Most of these transcripts are associated with repetitive SINE and LINE elements in the nucleolus (Figure 8(f)). Thus, although NAGs in these cancer lines are definitely transcribed, their proximity to the nucleolus does not facilitate inclusion of their transcripts in the nucleolar non-ribosomal RNA pool. These RNAs are transported out of the nucleolus and nucleolar periphery and become part of the general cellular RNA population. Reciprocally, many RNAs not transcribed in the nucleolus find their way there.

## Discussion

The identity and behavior of regions (NADs) and genes (NAGs) associated with the nucleolus has been the subject of a number of investigations [12,16,19]. These studies were carried out in a variety of human and mouse cell lines, as well as in *Arabidopsis thaliana*. As discussed in the Introduction, the conclusion reached on the basis of these studies was that for the most part the NAGs were packaged as heterochromatin and not expressed. Only a small number of NAGs appeared to be transcriptionally active; many of these coded for long non-coding RNA.

The individual studies describing these measurements typically addressed a limited number of cell types, different in each study, and with differences in methods of preparation of the nucleoli.

Here, we chose seven cancer cell lines from which nucleoli could be isolated by similar protocols (Methods), and their properties measured by identical procedures. Initially, as with previous studies, we used DNA sequence analysis of the purified nucleoli to identify sequences (NADs) enriched twofold or more in the nucleolar fraction. An average of 25–30% of the human genome was found to be NAD-associated, constituting between 7% (GM12878) and 43% (293T) of the genome. Of the seven cell lines, we found that four (Figure 2(d–g)) showed NAD distributions that varied among lines but had a general similarity in terms of NAD abundance to each other and to previously published results. These four cell-lines (which we grouped as Group 2) had relatively few NADs or NAGs, and largely did not share them with one another (Fig. S-1C, Figure 3(b), Fig. S-2, Table 2). In general, the appearance of the NAD maps resembled those reported for other cell lines. Surprisingly, the other three lines (293T, Jurkat-BCL2, and GM12878; Group 1) (Fig S-1B) had features more similar to one another and quite different from those of the other four lines.

One evident difference was in the chromosomal distribution of NADs. Aggregate maps of NAD distributions in Group 1 or Group 2 nucleoli (Fig. S-1 B, C) show that NAD sites in Group 1 nucleoli tend to cluster near telomeres, whereas those in Group 2 are more concentrated around centromeres. Most notable were the much higher abundance of NADs and NAGs in the Group 1 nucleoli, and the high concentrations of NADs on chromosome 19, 16, and 17. Equally important, these three cell lines shared a significant number of NAGs (Figure 3(a)) and contained within the NAG population many that are marked as being transcriptionally active (see below). These results appear to define two distinct groups of nucleoli in our population. The Group 1 nucleoli comprise a novel group with properties not previously described.

These differences were not reflected in nucleolar morphology: Immunofluorescence microscopy (Figure 1b) established that there is a high degree of variability in the number and size (ratio of nucleolar to nuclear volume) of nucleoli among the different cell lines we studied, but no obvious correlation with NAD or NAG content. It is well established that cancer cells generally have multiple nucleoli compared to non-cancer cells; the increased number of nucleoli is linked to higher levels of ribosome biogenesis, which they can achieve by the fusion of multiple small nucleolus to a large nucleolus [47].

The large number of NADs and NAGs in the Group 1 cells, and the contrast with Group 2 cells, led us to examine the Group 1 NAGs in greater detail. Previous studies [13,15] had shown that the majority of genes associated with the nucleolus were silenced, but only a few were expressed. We found that the Group 2 nucleoli behaved in this manner. The Group 1 nucleoli, however, were distinctly different. We assessed and compared the transcriptional activity of the NAGs in the two groups of nucleoli, using 293T and U2OS cells. The criteria for an active transcriptional state were ATAC-seq accessibility, H3K4me3, and presence of RNA Pol II at the NAG promoter. This revealed (Table 2) that in 293T cells 907 genes (13% of all NAGs) carried these three marks of transcriptional activity. Of these, 863 coded for a protein. In contrast, only about 1% of the 233 NAGs found in U2OS cells were similarly active.

A separate question is whether transcript from these genes can be detected. We found that over half (524/907) of the 293T genes carrying all three marks of gene activation are transcribed. Virtually all of these are protein coding genes, and the transcripts are exported to the nucleus (Table 2). In contrast, of the 233 NAGs in U2OS cells only three genes carried the activation marks and two were transcribed.

Strikingly, Gene Ontology analysis of the NAGs carrying activation marks in 293T nucleoli shows that they are principally involved in ribosome biosynthesis (Figures 6(e), 7), also seen in a similar analysis of NAGs common to all Group 1 nucleoli (Figure 3c). This group of genes does not appear to be especially concentrated on Chromosome 19, as shown in Gene Ontology studies of the Group 1 shared NAGs on that chromosome (Figure 4c). It should be noted that a relatively small number of NAGs are shared between Group 1 and Group 2 (Figure 3b, and Fig S-2) and that GO analysis of the latter suggests that unlike Group 1 NAGs, Group 2 NAGs are not devoted principally to ribosome biosynthesis.

Previous studies have found smaller numbers of NADs and NAGs in other cell lines. As noted, relatively few of the NAGs in those cells were found to be transcribed. The prevailing view is that such active genes are on the nucleolar surface and might be situated in small domains between the NADs, rather than within them [15]. We do not have direct information about the location within the nucleolus of the many active genes we identify in Group I nucleoli. The fact that essentially all these transcripts are released from the nucleolus suggests a location on the nucleolar periphery. However, the identification of H3K4Me3 marks within nucleoli could be consistent with active Pol II dependent transcription within the nucleolus as well (Figure 6(a,b,d)).

Quite recently, it has been reported that inhibition of RNA Pol II results in the formation of specific condensates that tether transcriptionally active chromatin to nucleoli. This suggests that the differences we see between the two groups of cell lines may reflect important metabolic differences between the two groups. One possibility is that 293T, Jurkat-BCL2, and GM12878 are lines arrested at earlier developmental stages. Perhaps, this explains the presence among their Group 1 NAGs of many genes related to ribosome biosynthesis; in Group 2 cells the demand for ribosomes may be less. Patterns of gene expression in nucleoli are a lot more varied than was anticipated, and further studies of other cells and cell lines will reveal still more novel patterns of activity reflecting the multiple roles of the nucleolus [48].

## Materials and methods

### Cell culture

Human cancer cell lines 293T (CRL-3216), Jurkat-BCL2 (CRL-2899), U2OS (HTB-96), SK-N-SH (HTB-11), Hela-S3 (CCL-2.2), and K562 (CCL-243) were obtained from ATCC, and GM12878 from Coriell Institute. Cells were cultured as per supplier protocol as detailed in Table 3.

**Table 3.** Summary of cell line and growth medium.

Cell Line	Growth Medium
Hela S3	F-12K (ATCC_30–2004) Base medium with 10% FBS
SK-N-Sh	IMDM (ATCC_30–2005) Base medium with 20% FBS
U-2OS	McCoy’s 5a (ATCC_30–2007) Base medium with 10% FBS
K-562	IMDM (ATCC_30–2005) Base medium with 10% FBS
BCL2-Jurkat	RPMI-1640 (ATCC_30–2001) Base medium with 10% FBS
GM12878	RPMI-1640 (ATCC_30–2001) with 2 mm L-Glutamate with 15% FBS (non-inactive)
293T	DMEM (ATCC_30–2002) Base medium with 10% FBS

### Antibodies, immunofluorescence microscopy, and CASFISH

All antibodies were obtained from Abcam if not mentioned otherwise. Detailed list of antibodies used in this study is provided in Table 4. Secondary antibodies, Goat anti-Rabbit Alexa Fluor® 488 (Abcam-Ab150077), and Goat anti-Mouse Alexa Fluor® 568 (Thermo Fisher Scientific, A11004) were used at the supplier’s recommended dilution.

**Table 4.** List of antibodies used in immunofluorescence and ChIP study.

Antibody	Target	Manufacturer and Catalog Number
Anti-Nucleophosmin	Nucleophosmin, nucleolus market	Abcam, ab10530
Anti-H3K9Me3	Histone H3 modification	Abcam, Ab8898
Anti-H3K36Me3	Histone H3 modification	Abcam, Ab9050
Anti-H3K9Ac	Histone H3 modification	Abcam, Ab177177
Anti-H3K4Me3	Histone H3 modification	Abcam, Ab8580
Anti-H3K27M3	Histone H3 modification	Abcam, Ab6002
Anti-RNA polymerase II CTD repeat YSPTSPS antibody	RNA polymerase 2	Abcam, ab26721
Anti-Lamin B1	Nuclear membrane marker	Abcam, Ab16048

For immunofluorescence study acquisition and visualization was performed using Zeiss LSM 780 Confocal Microscope. Images were accrued using 63X oil immersion objective using Zeiss Zen module.

For CASFISH, sgRNA targets were selected using the program Chop–Chop (https://chopchop.cbu.uib.no/) using randomly selected genes from chromosome-19 with wider coverage. sgRNA were synthesized by *in-vitro* transcription using Precision gRNA Synthesis Kit (Invitrogen) using manufacturer’s protocol. The purified sgRNA was quantified and labeled with Cy-5 using Label IT® Nucleic Acid Labeling Kits (Mirus Bio) using manufacturer’s protocols and purified using G50 microspin columns. Further, U2OS cells stably expressing NMP-mCherry under CMV promoter were transfected with *E. coli* expressed, dCas9 protein, sgRNA-Cy5, and incubated for 48 h under normal cell culture condition. Post incubation, the cells were thoroughly washed with PBS to remove excess labeled sgRNA and proteins. Acquisition and visualization of the cells were performed using Zeiss LSM 780 Confocal Microscope under similar condition mentioned above paragraph at 63X oil immersion objective.

### Nucleolus isolation

Nucleolus was isolated using previously published protocols [15]. Briefly, cells (10^8^) were grown to ~80% confluency and harvested. Cell pellet was resuspended in high magnesium buffer [HM: 10 mm HEPES pH 7.5, 1.5 mm MgCl_2_, 1 mm dithiothreitol (DTT), 10 mm KCl, and protease inhibitors cocktail] and incubated on ice. Post-incubation cells were ruptured in Dounce tissue homogenizer. Homogenized cells were centrifuged at 218 g for 5 min at 4°C. The pellet was resuspended in 3 ml S1 solution (0.23 M Sucrose, 10 mm MgCl_2_) and layered over 3 ml of S2 solution (0.35 M Sucrose, 0.05 mm MgCl_2_) and centrifuged at 1430 g for 5 min at 4°C. The resulting pallet was resuspended in 3 ml of S2 solution. Intact Nucleoli were released from this suspension by sonication on ice (10 bursts of 10s each at full power) using a Soniprep 150 (MSE) with a fine probe. The sonicated sample was then layered over 3 ml of S3 solution (0.88 M Sucrose, 0.5 mm MgCl2) and centrifuge at 3000 g for 10 min at 4°C, resulting in the precipitation of purified nucleoli. These nucleoli were finally suspended in 0.5 ml of S2 solution, followed by centrifugation at 1430 g for 5 min at 4°C for final purification.

Final purified nucleoli were resuspended in 0.5 ml S2 and used immediately for DNA or RNA extraction. For long-term storage, nucleoli were resuspended in buffer S2 and stored at −80°C until further use.

### Nuclear and nucleolar DNA library preparation and alignment

For nucleolar DNA library preparation 0.5 ml of suspended nucleoli was used for DNA isolation using PureLink genomic DNA isolation Kit (Thermo Fisher Scientific, Catalog – K-1820-02), and 250 bp indexed DNA libraries were prepared in triplicate using MicroPlex-Library Kit – V2 (Diagenode, Catalog – C05010012) as per manufacturer’s protocol. For control (input), whole cell DNA libraries were made according to protocol described above using 10^6^ freshly harvested cells from the same cell line. The indexed libraries were sequenced in the HiSeq 3000 sequencer (Illumina).

Whole cell/nuclear (N) and nucleolar (No) sequence libraries from different cell lines (input) and corresponding nucleolar libraries containing millions of reads were prepared, and quality was assessed using FastQC [49] The adapters were trimmed using Cutadapt [50]. The remaining reads were mapped to the human genome (hg19) using bowtie2 [51] and no mismatches were allowed. The same files were obtained after the alignment, converted into bam files, sorted, and indexed using samtools [52]. The reads were quantified into 100kb size bins using bedtools software [53]. The read densities of the nuclear (N) and nucleolar DNA (No) were compared. Genomic regions showing two-fold or greater nucleolar to nuclear (No/N) enrichment were considered as nuclear associated domains (NADs). The NADs were annotated, and nucleolar associated genes (NAGs) were identified using ChIPpeakAnno [54]. The Biomart package in R [55] was used to retrieve the annotation data human GRCh37. The gene ontology (GO) analysis was carried out using Web-based Gene Set Analysis Toolkit (webgestalt) [56].

### Nucleolar ATAC-seq and data analysis

ATAC sequencing was performed on 293T and U2OS nucleoli purified from 10^6^ cells, by modification of the protocol used for intact cells, using an ATAC-seq Kit (Active motif – Catalog 53,150). The indexed libraries were sequenced by ATAC seq services from Active motif (Catalog – 25,079).

The paired-end sequencing reads generated by Illumina sequencing were mapped to the genome using the BWA algorithm with default settings [57]. Alignment information for each read is stored in the BAM format. Only reads that pass Illumina’s purity filter, align with no more than two mismatches, and map uniquely to the genome were used in the subsequent analysis. Genomic regions with high levels of transposition/tagging events were determined using the MACS2 peak calling algorithm [58]. Since both reads (tags) from paired-end sequencing represent transposition events, both reads were used for peak-calling but treated as single, independent reads. To compare peak metrics between two or more samples, overlapping intervals were grouped into ‘Merged Regions’, which are defined by the start coordinate of the most upstream interval and the end coordinate of the most downstream interval. After defining the Intervals and Merged Regions, their genomic locations along with their proximities to gene annotations and other genomic features were determined.

### Nucleolar ChIP-seq and data analysis

Nucleolar DNA ChiP was performed on purified nucleoli from U2OS and 293T cells using iDeal ChIP-seq Kit for Histones (Diagenode, Catalog – C01010051) followed by ChIP indexed libraries in triplicate using MicroPlex-Library Kit-V2 (Diagenode, Catalog – C05010012) as per manufacturer’s protocol. Total nucleolar DNA libraries were used as control (input). The indexed libraries were sequenced in the HiSeq 3000 sequencer (Illumina).

Raw reads for different histone marks (H3K4me3 (Abcam – ab8580), H3K9me3 (Abcam – ab8898), H3K27me3 (Abcam – ab6002)) and RNA Pol II (Abcam – ab5095) were mapped to human genome 19 (hg19) using Bowtie2 [51]. Post-alignment bam files were converted to bam using Samtools [52]. The bam files representing the read counts were normalized and visualized using IGV genome browser [20]. Positive peak signals were identified using the Model-based Analysis for ChIP-Seq (MACS) program [58]. The bam files for biological replicates were merged using Samtools for quantitative analysis. ChIP-seq signal around the transcription start site (TSS) was analyzed with the ngs.plot tool [59]. The peaks were annotated using ChIPseeker package in R [60].

### Nucleolar RNA-seq and data analysis

Indexed cDNA libraries of nucleolar mRNA purified from five different purified nucleolar samples (293T, GM12878, K562, Sn-K-SH, and U2OS) were prepared in triplicate by mRNA enrichment and rRNA Depletion using NEBNext® Ultra™ II RNA Library Prep Kit for Illumina (NEB E7775) and NEBNext® rRNA Depletion Kit (Human/Mouse/Rat) (NEB E6350) following manufacturer’s protocol. Final Indexed cDNA libraries were prepared using MicroPlex-Library Kit – V2 (Diagenode, Catalog – C05010012). For control, corresponding total cellular mRNAs were prepared and used using the above protocol. The indexed libraries were sequenced in the HiSeq 3000 sequencer (Illumina).

Paired-end sequencing files were generated by Illumina for nucleolar and nuclear mRNA. The quality of reads was assessed using FastQC [49]. The ribosomal RNA was filtered using SortMeRNA tool [61]. Adapters were trimmed using Trimmomatic [62]. The trimmed reads were mapped to the human genome (hg19) using default parameters of STAR (Spliced Transcripts Alignment to a Reference, version 2.4.0.1) tool [63]. The read counts were normalized, and the differential gene expression analysis was done using DESeq2 package in R [64].

## Supplementary Material

Supporting Information.docx

## Data Availability

All data are contained in the manuscript as plotted graphs or representative\image files. Source files in TIFF/JPG and .cvs format or excel files are available from the corresponding author (G.F.) upon request. Raw sequencing file data of DNAseq, RNAseq, and CHiPseq will be submitted to NCBI (https://www.ncbi.nlm) for public release once the manuscript is accepted for publication.

## Significant statement

Nucleoli of certain cancer cell lines unexpectedly contain many non-ribosomal genes actively transcribed by RNA polymerase II. Many of these genes are expressed in all those lines. This behavior might be a characteristic of certain cancer cells.
