# Supplementary material for: RNA Pol-II transcripts in nucleolar associated domains of cancer cell nucleoli
Source: Nucleus. 2025 Feb 23;16(1):2468597. doi: 10.1080/19491034.2025.2468597 (PMC11849958; doi:10.1080/19491034.2025.2468597)
Supplement: Supporting Information.docx [file KNCL_A_2468597_SM6394.docx]

**Supporting Information:**

**S-1**


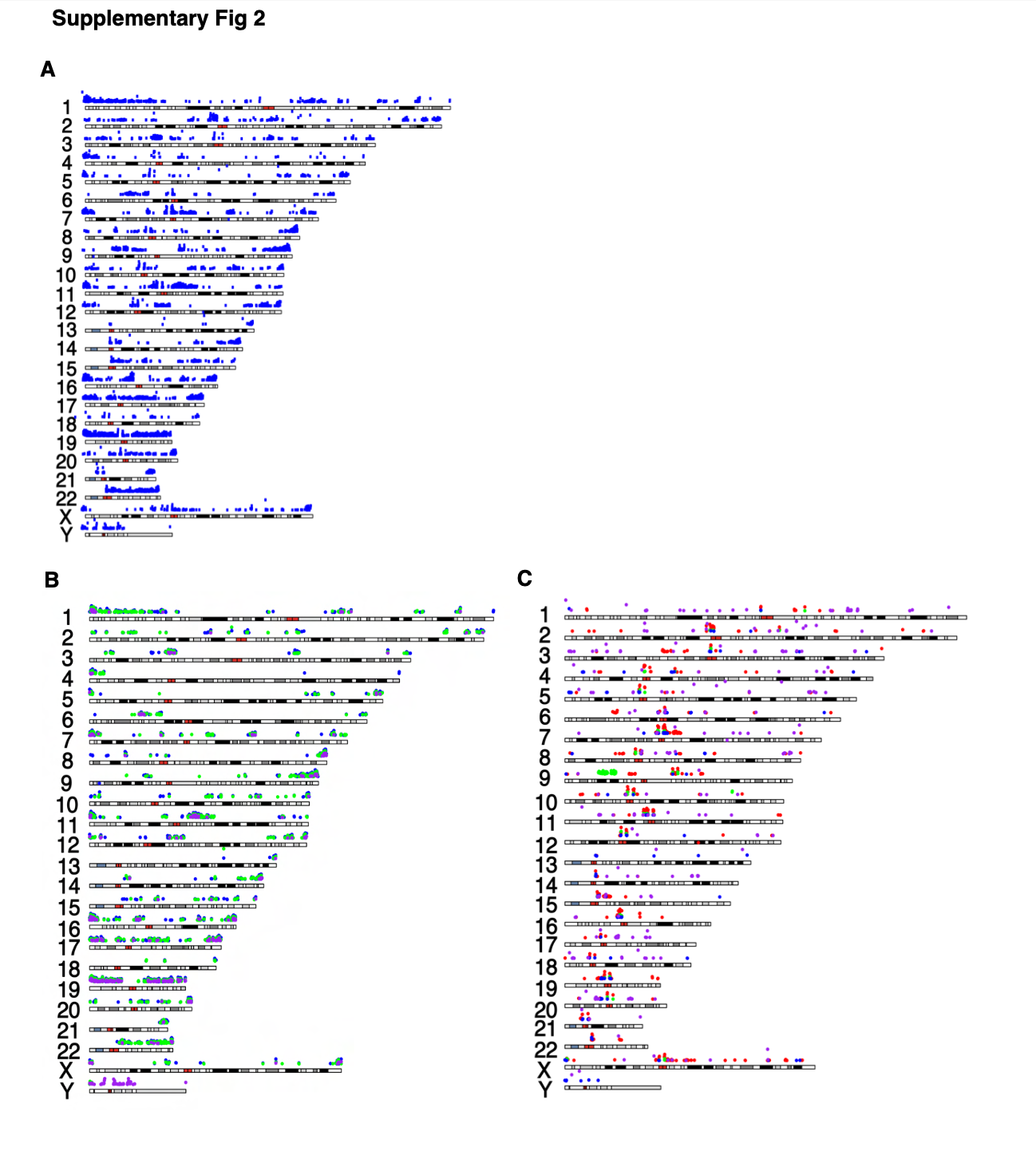
 **S-1**: Overall distribution of NADs in all cell-lines A) Karyotypic-distribution of NADs in class 1 (293T(blue), GM12878 (purple) and Jurkat-BCl2 (green) B) Class 2 (Hela-S3 (red), K562 (green), SK-N-SH (blue) and U2OS (purple)) cell lines.

**S-2**


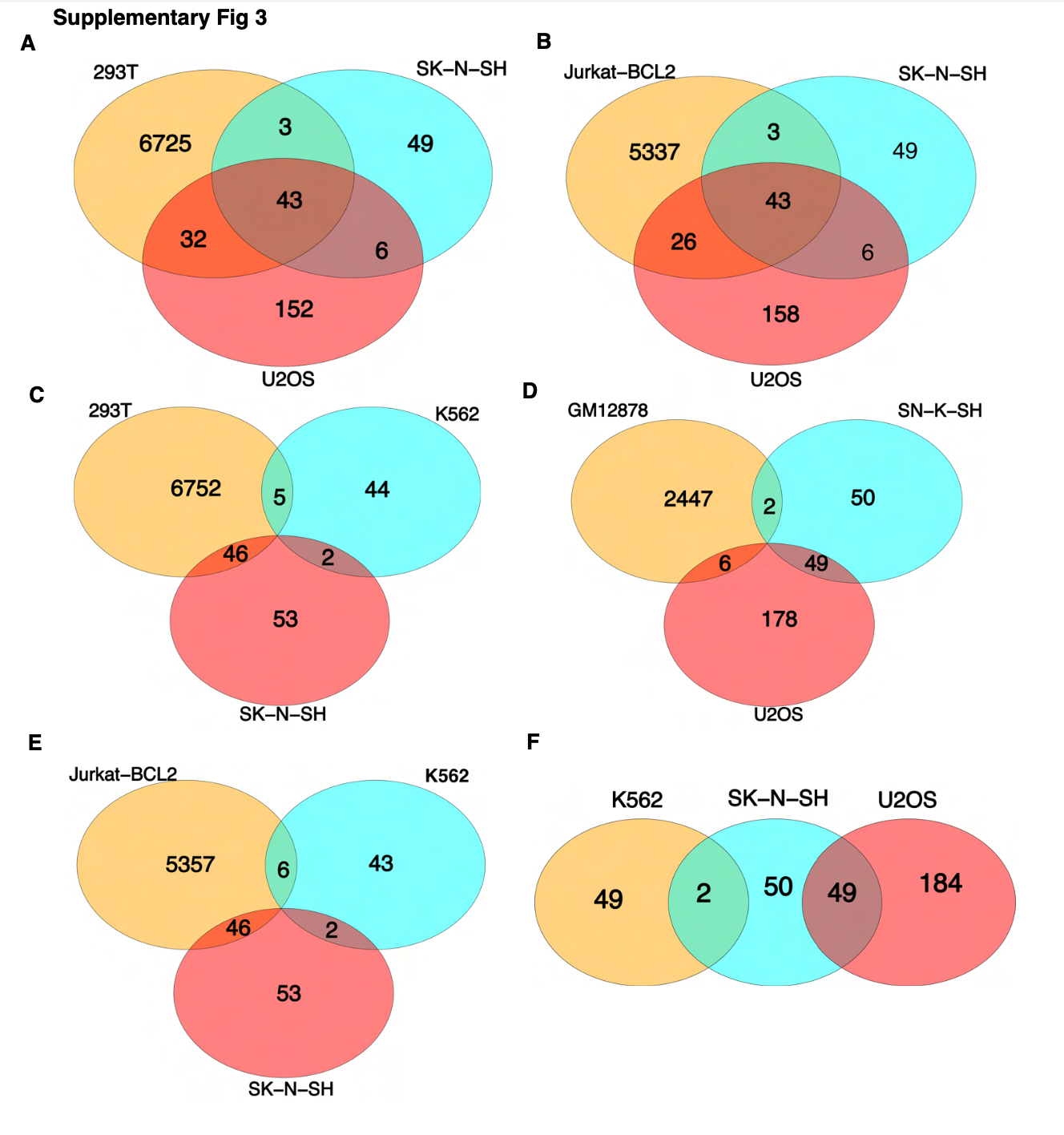


**S-2**: Venn diagrams showing overlap of NAGs among different cell lines.

**S-3**


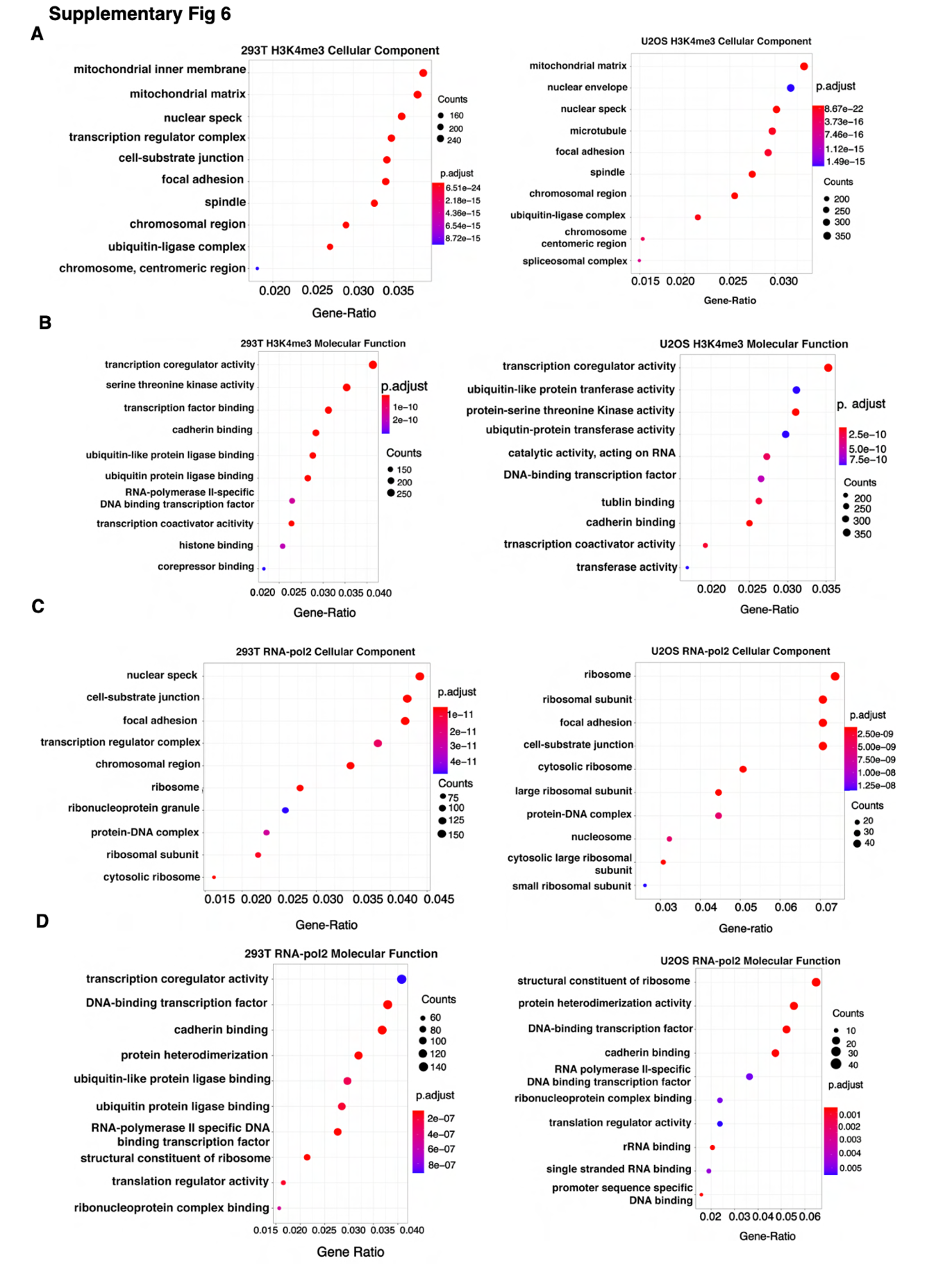


**S-3**: Functional characterization of genes associated with H3K4me3 and RNA pol2 in 293T and U2OS cell lines.

**S-4**


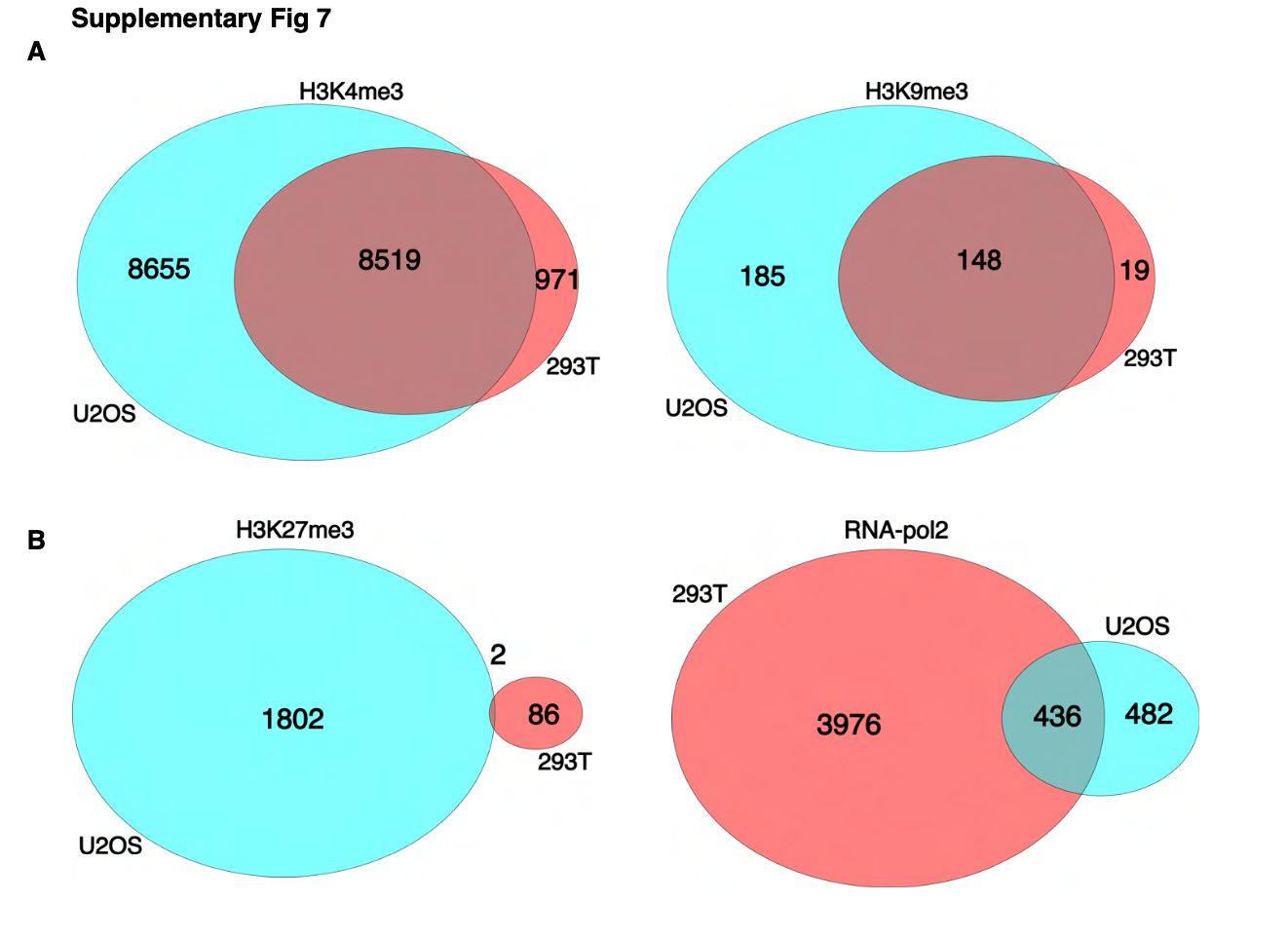


S-4. Venn diagram showing overlapping peaks for histone modifications and RNA pol2 in 293T and U2OS cells.

**S-5**


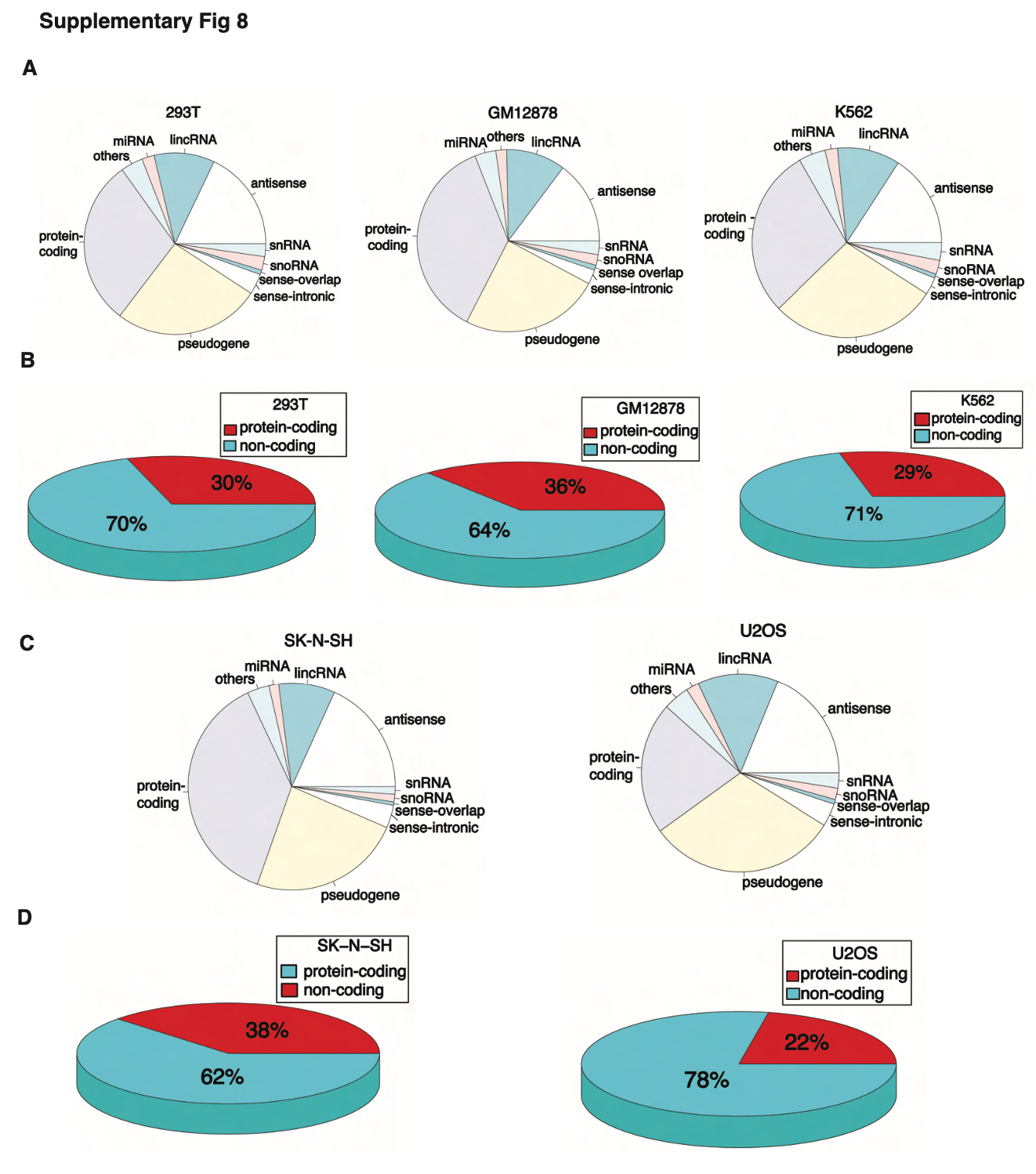


S-5: The percentage of protein-coding and non-coding genes in the nucleolus across different cell lines (293T, GM12878, K562, SK-N-SH and U2OS).

S-6


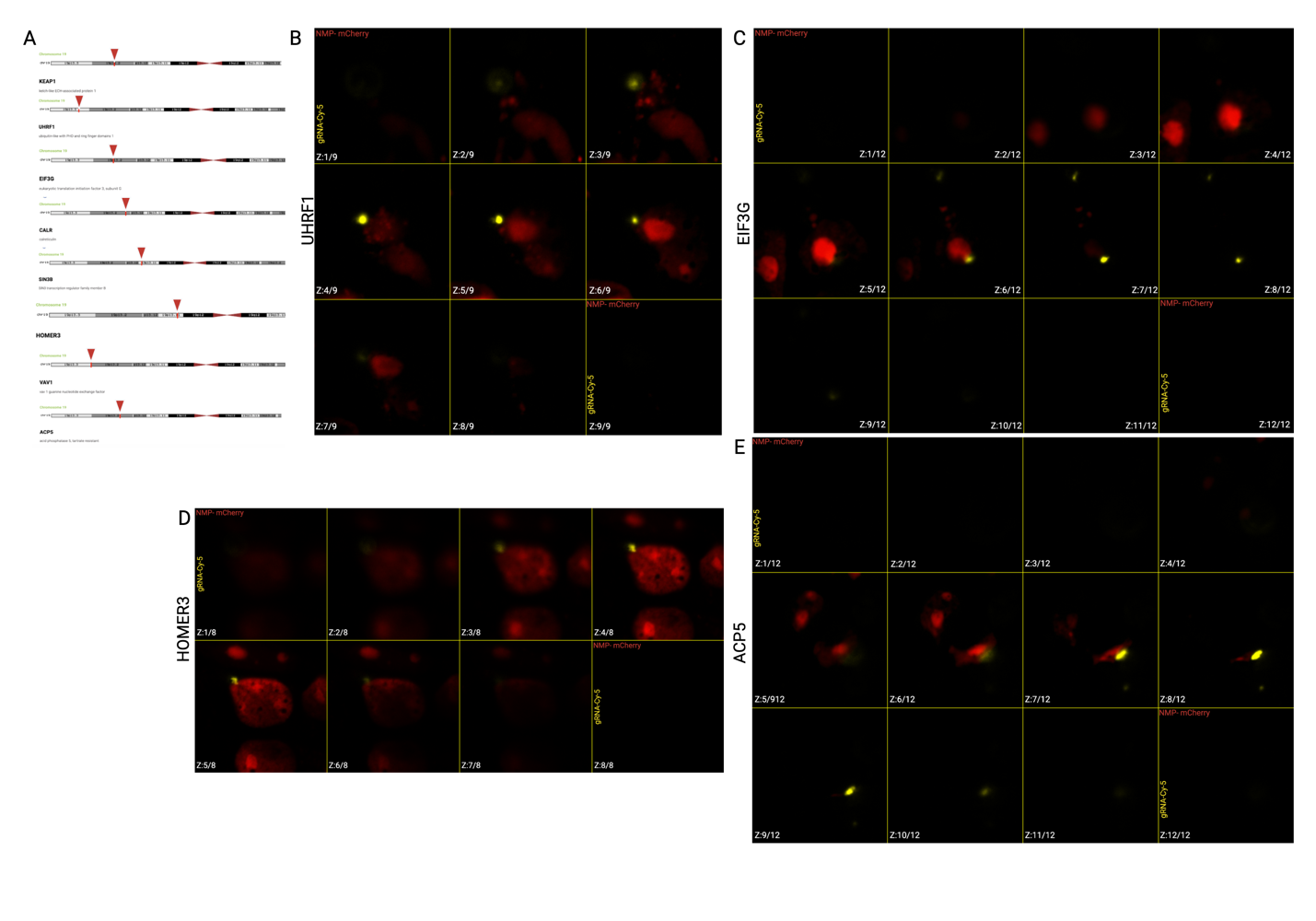


S-6: Detection of Chromosome -19 genes in the nucleolus and nucleolar periphery using CASFISH. Stable constructs of U2OS cell lines were made by transfecting the cells with NMP-1-mCherry under CMV promoter. Next gRNAs constructs were made targeting randomly selected genes in Chromosome-19 as shown in Figure A with the genes location in chromosome-19 marked with red arrow, using invitro transcription. The purified gRNAs were labeled with Cy-5 and further purified to get rid of unbound labels.

The U2OS-NMP-mCherry cells were co-transfected with dead Cas9, dCas9 protein, and gRNA-Cy-5 in equimolar concentration. After 48hrs incubation, live cell imaging was perfumed at 63X resolution. Among the 8 target genes selected for Chromosome-19 markers, 4 genes, namely, UHRF1(Figure B), EIF3G (Figure C), HOMER3 (Figure D) and ACP5 (Figure E), showed localization to nucleolar periphery or vicinity, signifying the proximity of Chromosome-19 to the nucleolus.

S-7


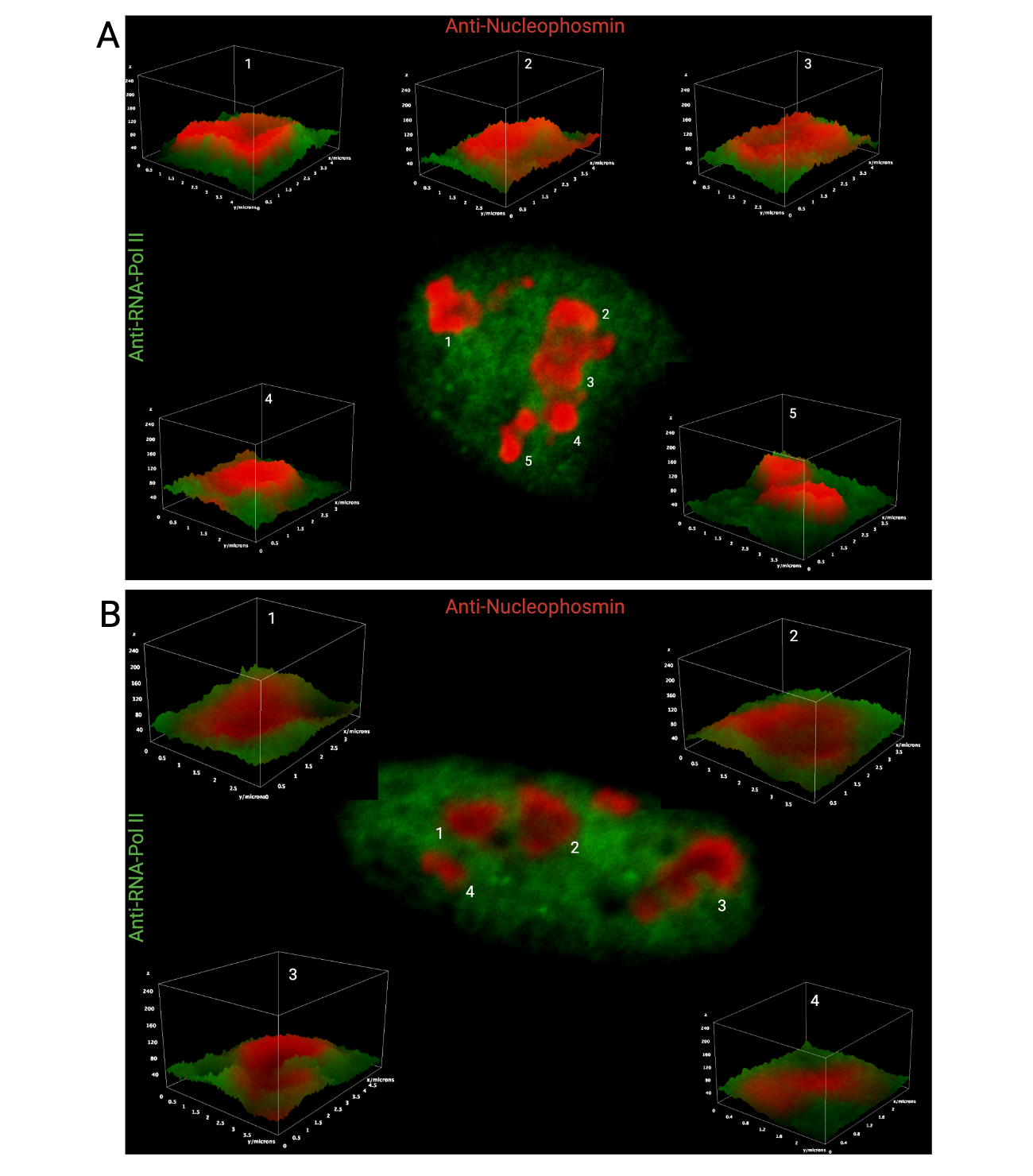


S-7: Immunofluorescence of U2OS cell with anti RNA pol II and anti- Nucleophosmin antibodies showing the presence of RNA pol II within and surrounding the Nucleolus boundary. Surface plot was analyzed for individual Nucleolus (inset for panel A and B) for single cells as numbered respectively, and distribution of both antibody targets are shown. Yellow peaks designate the presence of RNA- pol II with in the nucleolar boundary and inside the nucleolus.
